# K-RAS4A: Lead or Supporting Role in Cancer Biology?

**DOI:** 10.3389/fmolb.2021.729830

**Published:** 2021-09-15

**Authors:** Veronica Aran

**Affiliations:** Laboratorio de Biomedicina Do Cérebro, Instituto Estadual Do Cérebro Paulo Niemeyer, Rio de Janeiro, Brazil

**Keywords:** K-ras, K-Ras4A, K-Ras4B, alternative splicing, cancer

## Abstract

The RAS oncogene is one of the most frequently mutated genes in human cancer, with K-RAS having a leading role in tumorigenesis. K-RAS undergoes alternative splicing, and as a result its transcript generates two gene products K-RAS4A and K-RAS4B, which are affected by the same oncogenic mutations, are highly homologous, and are expressed in a variety of human tissues at different levels. In addition, both isoforms localise to the plasma membrane by distinct targeting motifs. While some evidence suggests nonredundant functions for both splice variants, most work to date has focused on K-RAS4B, or even just K-RAS (i.e., without differentiating between the splice variants). This review aims to address the most relevant evidence published regarding K-RAS4A and to discuss if this “minor” isoform could also play a leading role in cancer, concluding that a significant body of evidence supports a leading role rather than a supporting (or secondary) role for K-RAS4A in cancer biology.

## Introduction

The importance of gene alternative splicing has been well documented. This conserved biological process occurs when a single gene produces different mRNA transcripts, thus helping to contribute to the formation of a vast transcriptome and proteome (Kelemen et al., 2013). This process generates protein diversity, as a single gene can result in the production of different variants of a protein, which may exhibit differential tissue expression (Sorek and Amitai 2001). In summary, alternative splicing results in different: 1) protein function; 2) tissue expression; 3) localisation; enzymatic activities; and 4) protein-protein interactions (Kelemen et al., 2013). The differences between splice variants are of pharmaceutical importance since they may contribute to variable treatment responses.

**FIGURE 1:**
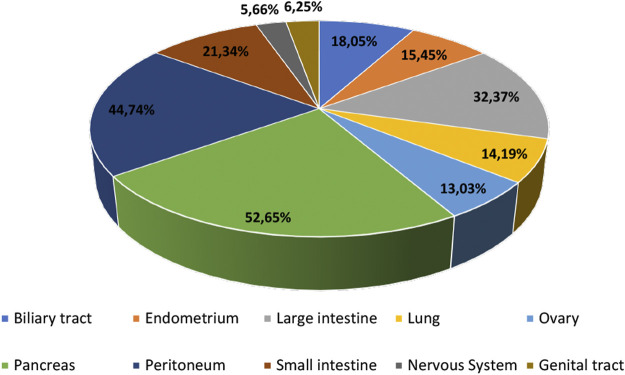
Most frequent human tissues affected by K-RAS mutations based on the COSMIC database, v94 (data obtained in May 2021).

There are three RAS genes encoding four isoforms, which are ubiquitously expressed in human cells and share 82–90% sequence homology. These four isoforms are H-RAS, N-RAS, K-RAS4A and K-RAS4B (Cox and Der 2010). *RAS* mutations are frequently found in cancer (∼24% of all cancers) (Stalnecker and Der 2020), where the K-RAS gene is mutated in approximately 17% of all cancer types (46,213 mutant samples/272047 samples tested), N-RAS gene is mutated in ∼5.1% (7,926 mutant samples/154172 samples tested), and H-RAS in ∼2.3% (2,404 mutant samples/106318 samples tested) (as reported in the Catalog of Somatic Mutated in Cancer, COSMIC database, v94, in August 2021). RAS mutations are crucial for personalised medicine since they can direct targeted therapies and serve as diagnostic and prognostic markers for different cancers (Murugan et al., 2019). In fact, K-RAS mutations were considered adverse prognostic factors and indicators of EGFR-targeted therapy resistance in certain cancer types such as lung and colorectal (Pao et al., 2005; Marks et al., 2008; Normanno et al., 2009). Figure 1 summarizes some of the most frequently K-RAS mutated tissues based on the COSMIC database (searched in COSMIC database, v94, in May 2021).

The discovery over 35 years ago (McGrath et al., 1983; Shimizu et al., 1983) of the fourth exons 4A and 4B resulted in the identification of the existence of two protein isoforms, K-RAS4A and K-RAS4B [189 and 188 amino acids (aa), respectively]. The 21-kDa RAS gene products shares 100% sequence homology in the first 86 aa residues among different RAS isoforms (K-, N- and H-RAS) (Messina et al., 2019). The RAS G domain comprises the first 165 aa, representing the catalytic and switching region where the exchange between GDP/GTP occurs. It is also the domain to which different effectors, exchange factors, and GTPase-activating proteins (GAPs) bind. K-RAS plays an essential role in mouse embryonic development (Koera et al., 1997; Hobbs, Der, and Rossman 2016), whereas K-RAS4A, H-RAS and N-RAS expression is dispensable for mouse development (Esteban et al., 2001; Plowman et al., 2003). Unlike K-RAS4A, K-RAS4B has been heavily researched.

Mutations that activate K-RAS usually affect codons 12, 13, and 61 (the majority being missense substitutions), which are common to both genes, thus rendering both oncogenic (Capon et al., 1983). The biological relevance of the alternative splicing of K-RAS has never been fully elucidated. Most studies have concentrated their attention on K-RAS4B rather than K-RAS4A. For example, while a *Pubmed* search on “K-RAS” or “KRAS” yields 21,408 results, a search on “K-RAS4A” or “KRAS4A”or “K-Ras4A” or “KRas4A” or “K-Ras4a” or “KRas4a” yields 54 results and a search on “K-RAS4B” or “KRAS4B″or “K-Ras4B” or “KRas4B” or “K-Ras4B” or “K-Ras4b” or “KRas4b” yields 213 results (all searched on May 20, 2021). This finding suggests that most studies have not discriminated by K-RAS isoform. Nevertheless, the two splice variants exhibit differential tissue expression (Newlaczyl et al., 2017). Therefore, the present review aims to improve the general understanding of each isoform by describing previous work an discussing potential roles of K-RAS4A in cancer.

## K-RAS4A Versus K-RAS4B: Structure and Signalling

It is well stablished that RAS isoforms exhibit distinct biological activities and subcellular localisations that depend mainly on the interaction between the C-terminal hypervariable region (HVR) and host membranes (Hancock 2003; Laude and Prior 2008). The HVR region is composed of a linker domain comprising aa 166–178/179, and a targeting domain comprising aa 179/180–189/188, which undergoes posttranslational modifications that mediate membrane binding. The HVR contains a C-terminal CAAX (CAAX motif) sequence, which is modified posttranslationally (Wright and Philips 2006). The C-terminal cysteine is farnesylated for weak membrane interaction; further membrane binding stabilisation requires a second signal within the HVR region (Hancock et al., 1991). For K-RAS4B, this signal is electrostatic (i.e., six contiguous lysines), whereas for the other RAS isoforms (K-RAS4A, H-RAS and N-RAS), this stabilisation is mediated by palmitoylation (Hancock et al., 1990). The isoform H-RAS contains two palmitoylation sites within the HVR region, whereas N-RAS and K-RAS4A are monopalmitoylated (Zhou et al., 2018). Additionally, K-RAS4B displays a unique feature, a phosphorylation site (aa S181) that behaves as an electrostatic farnesyl switch, inducing K-RAS4B translocation from the plasma membrane to other endomembrane compartments (Barcelo et al., 2014). The different posttranslational modifications that occur in the RAS C-terminal region were, and still are, considered potential targets for anti-cancer therapies despite the failure of farnesyltransferase inhibitors in the past (James, Goldstein, and Brown 1996; Konstantinopoulos et al., 2007; Ahearn et al., 2018).

RAS interaction with the plasma membrane is required for its function. K-RAS4A and K-RAS4B differ mainly in their C-terminal regions (Laude and Prior 2008; Tsai et al., 2015), which in the case of K-RAS4A, contains a site of palmitoylation and a bipartite polybasic region able to independently deliver K-RAS4A to the plasma membrane (Laude and Prior 2008; Tsai et al., 2015; Zhao et al., 2015). This indicates that, compared to other RAS proteins, K-RAS4A is the only one harbouring a dual membrane-targeting motif and that K-RAS4B is more positively charged and less hydrophobic than K-RAS4A. It has been proposed that the bipartite polybasic region alongside the monopalmitoylation and farnesylation of K-RAS4A may affect its function and expression, in addition to place this variant between K-RAS4B and N-RAS in terms of protein similarities (Laude and Prior 2008; Nussinov et al., 2016).

Structural analysis using atom molecular dynamics simulations investigated K-RAS4A placement at membranes that contain anionic lipids (POPS or PIP2) (Li and Buck 2017). This study demonstrated that K-RAS4A prefers different orientations at the membrane, where both its topology and the electrostatic interaction between its charged residues and the anionic lipids influence its orientation (Li and Buck 2017). Hancock and others reported that inhibition of acid sphingomyelinase mislocalises K-RAS4A and K-RAS4B from the plasma membrane to the endomembrane and blocks their nanoclustering, thus suggesting that an indirect inhibitor of sphingomyelinase could serve as a potential anti-K-RAS agent (Cho et al., 2016).

The protein conformations of K-RAS4A and K-RAS4B have also been compared by all-atom molecular dynamics simulations to identify isoform-specific differences. The results suggested that the catalytic domain of GDP-bound K-RAS4A differs from that of K-RAS4B by presenting a more exposed nucleotide binding pocket, also showing distinct dynamic fluctuations in switch I and II regions, which could affect the interaction between the catalytic domain and downstream effectors (Chakrabarti et al., 2016).

All four RAS isoforms have been shown to possess different biological activities and effector signalling. At least 11 different RAS effector families have been described, which drive distinct signalling cascades (Hobbs et al., 2016). Although all RAS proteins can differentially activate the Raf-MEK-ERK signalling pathway and affect cell phenotype *in vitro*, K-RAS4A and K-RAS4B have been shown to differentially affect Raf-1 (Voice et al., 1999). Furthermore, application of stable isotope labelling with amino acids in cell culture (SILAC) and affinity-purification mass spectrometry (AP-MS) to characterize the nucleotide-dependent protein interactomes of K-RAS4A and K-RAS4B revealed novel interactomes for each variant, with comparable numbers of interacting proteins for both wildtype and mutant versions of each splice variant (Zhang et al., 2018). Zhang and others described that K-RAS4A interacts with Raf-1 with higher affinity than K-RAS4B, leading to increased RAF1-1MEK-ERK signalling cascade, and that K-RAS4A showed increased anchorage-independent growth in assays that compared K-RAS4A- and K-RAS4B-transformed NIH 3T3 cells (Zhang et al., 2018). Interestingly, Bigenzahn and others performed proteomic analysis using K-562 chronic myeloid leukaemia cell lines. They reported that, while the two RAS isoforms share 28 interactors, they also each have distinct interactomes, with K-RAS4A specifically binding to fewer proteins than K-RAS4B (15 proteins versus 29, respectively) (Bigenzahn et al., 2018). Cumulatively, these findings suggest a certain degree of functional overlap and also raise the possibility that the splice variants cooperate with each another or compensate for each other’s function, depending on the cell type and intracellular pathway involved.

K-RAS4A protein was identified as a defattyacylation substrate of SIRT2, a member of the sirtuin family of protein lysine deacylases (Jing et al., 2017). Through biochemical and cell biology approaches, Jing and others found that K-RAS4A is fatty acylated on lysine residues at its C-terminal HVR, and that SIRT2 removes lysine fatty acylation from K-RAS4A, resulting in increased endomembrane localisation, interaction with A-Raf, and in turn enhanced K-RAS4A transforming activity (Jing et al., 2017). Thus, the study of small molecules that could inhibit the defatty-acylation activity of sirtuins may have therapeutic potential. Spiegelman and others developed a SIRT2 inhibitor, named JH-T4, which was the first such inhibitor to enhance K-RAS4A lysine fatty acylation *in vitro* (Spiegelman et al., 2019). Although JH-T4 showed anti-cancer effects in cancer cells, it was also toxic to normal cells, suggesting a lack of cancer cell selectivity (Spiegelman et al., 2019). Thus, JH-T4, while potentially promising, awaits further improvements that may enhance its cancer cell selectivity.

**FIGURE 2:**
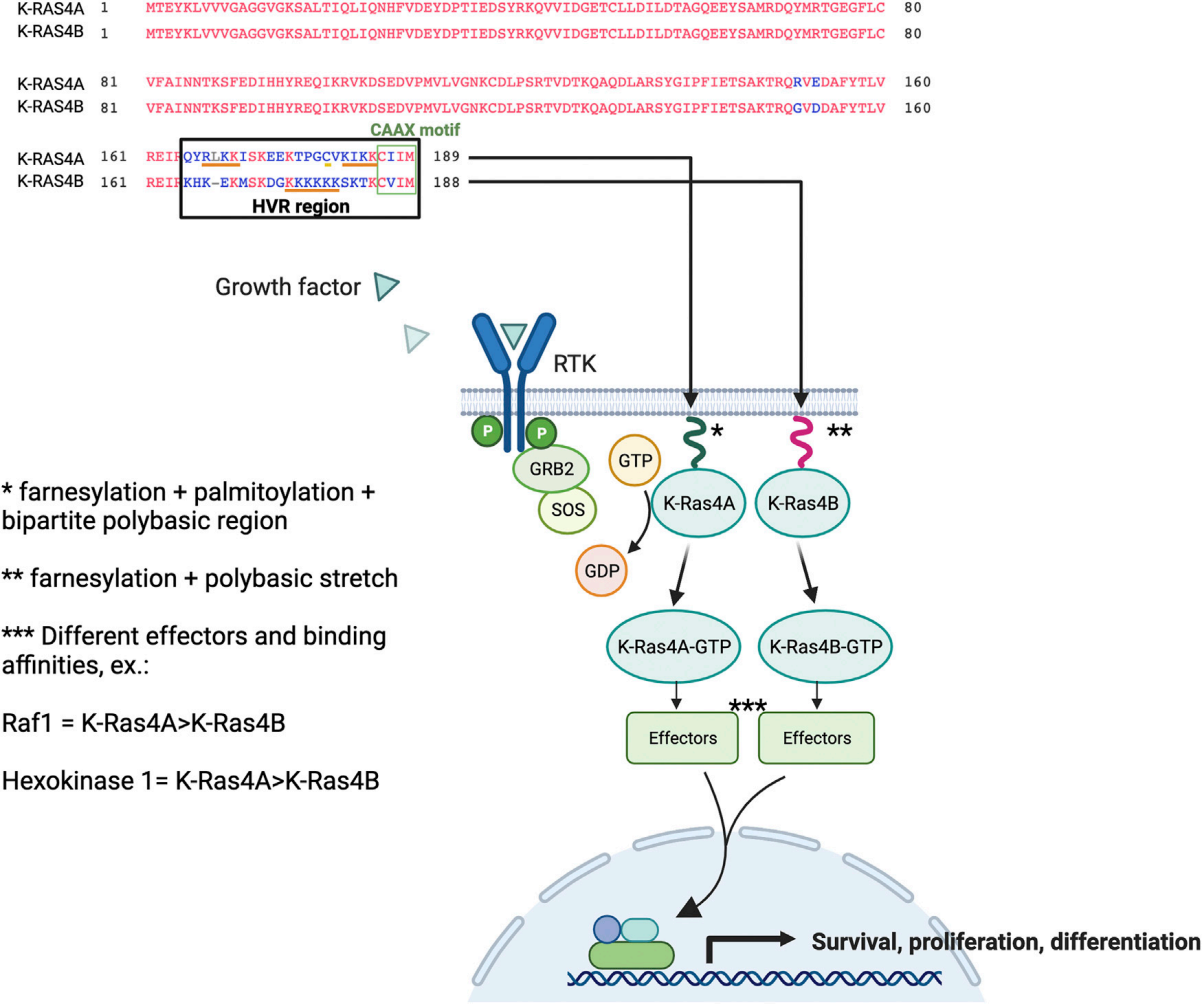
K-RAS4A versus K-RAS4B protein sequence and signalling. RAS proteins contain a G domain (residues 1–165) responsible for the catalytic and switching portion of the protein that interacts with GDP/GTP, exchange factors, and GTPase-activating proteins (GAPs). The less homologous part corresponds to the hypervariable region (HVR) domains (final 23/24 residues). RAS are farnesylated on their C-terminal cysteine residue (CAAX motif), undergo AAX proteolysis and receive carboxyl methylation at the C-terminal prenylcysteine to allow the first step in membrane binding. A second motif improves this weak binding, which is a hexa-lysine polybasic stretch (residues 175–180) that interacts electrostatically with membranes in the case of K-RAS4B. Whereas, K-RAS4A membrane binding is stabilised by a monopalmitoylation site (residue 180), whereas this site is absent in the isoform K-RAS4B (Hancock et al., 1991). The KIKK motif (residues 182–185) was shown to be an additional membrane-targeting motif for K-RAS4A (Zhao et al., 2015), in addition to other basic motif corresponding to RLKK (residues 167–170) (Tsai et al., 2015). The binding of growth factors to the extracellular regions of receptor tyrosine kinases (RTKs) initiates the signal that will lead to the activation of RAS proteins downstream of the receptor. The isoforms can bind to a variety of effectors with variable affinities where, for example, hexokinase 1 was shown to bind to K-RAS4A with higher affinity than to K-RAS4B; the same was observed for Raf1 (Zhang et al., 2018; Amendola et al., 2019). Part of the figure was built and adapted from the “Ras Pathway” template, by BioRender.com.

Collectively, the studies suggest that RAS effector pathways may be differentially impacted by RAS structural conformation, localisation to membranes, and isoform-specific binding affinities, which may lead to variable signalling outputs. Figure 2 compares the K-RAS4A and K-RAS4B protein sequences, highlighting the important residues for membrane binding, and also the simplified schematic representation of the RAS pathway indicates that each isoform has its own binding affinities for different effectors, which may result in a variety of cell responses.

## Comparison of K-RAS4A and K-RAS4B Tissue Expression Profiles

Profiling of K-RAS splice variant expression have shown that K-RAS4A and K-RAS4B expression levels differ across tissues. The K-RAS4A/4B expression ratio varies according to normal versus tumour tissues, as well as by tumour type analysed (e.g. lung, pancreas and colorectal cancer) (Plowman et al., 2003; Abubaker et al., 2009; Aran et al., 2018). For example, in patients with non-small cell lung cancer (NSCLC), K-RAS4B mRNA showed higher expression than K-RAS4A (Aran et al., 2018). In contrast, similar splice variant levels were reported in the colon (Pells et al., 1997). When the gene expression profiles of each RAS isoform were characterized in a full developmental time course mouse tissue panel, K-RAS4B expression was frequently higher than K-RAS4A (Newlaczyl et al., 2017). The findings suggested that K-RAS4A is the most dynamically regulated RAS isoform (upregulated in pre-term in stomach, intestine, kidney and heart) (Newlaczyl et al., 2017).

A quantitative RT-PCR assay has been developed to detect the splice junction region and thus measure variant expression in human cancer cell lines (Tsai et al., 2015). Of the 30 cell lines tested, the isoform K-RAS4A was expressed in all of them; with similar levels to that of K-RAS4B detected in 17 human colorectal tumours. Analysis with splice variant-specific antibodies supported this finding (Tsai et al., 2015). K-RAS4A showed higher expression in colon cancer and melanoma cell lines than in other cell lines tested (Tsai et al., 2015). Furthermore, there were no significant differences in the relative abundance of the two K-RAS mRNAs among cells that harboured wildtype versus mutant K-RAS. Another study showed that K-RAS4A was found to be expressed in both human renal cell carcinomas and human renal cell carcinoma cells lines, with its upregulation sensitive to aldosterone (King et al., 2014).

As previously mentioned, the K-RAS4A HVR sequence shares similarities with those of K-RAS4B and N-RAS. Nussinov and colleagues proposed that the N-RAS-like state of K-RAS4A (i.e., palmitoylated and farnesylated) could influence its high expression in melanoma, and that the K-RAS4B-like state of K-RAS4A (i.e., farnesylated) could contribute to the high expression levels seen in colon cancer (Nussinov et al., 2016).

Regarding benign tumour tissues, Shahrabi-Farahani and colleagues reported that during the proliferative phase of the menstrual cycle, K-RAS4A mRNA was upregulated (2.7-fold higher) in eutopic endometrium in patients with endometriosis compared to controls (Shahrabi-Farahani et al., 2015), whereas no significant correlation was observed between K-RAS4B and the different menstrual cycle phases (Farahani et al., 2015). Shahrabi-Farahani and colleagues proposed that increasing the K-RAS4A\4B ratio could affect the equilibrium between proliferation and apoptosis, two processes that are responsible for maintaining a normal eutopic endometrium, thus leading to the proliferative phase defect seen in patients with endometriosis. Furthermore, expression of both splice variants was also detected in patients with leiomyoma (i.e., uterine tumours originating from smooth muscle cells) (Zolfaghari et al., 2017).

## Possible Roles for K-RAS4A in Tumorigenesis

Different roles have been attributed to K-RAS4A. Studies of embryonic stem cells have suggested that K-RAS4A promotes apoptosis while K-RAS4B inhibits it, and that the K-RAS4A/4B isoform ratio regulates tumorigenesis by influencing stem cell differentiation and survival (Plowman et al., 2006). In addition, K-RAS4A was recently shown to be enriched in cancer stem-like cells under hypoxia conditions, whereas K-RAS4B was mainly induced by ER stress (Chen et al., 2021). Chen and colleagues also suggested that K-RAS4A splicing could be controlled by the DCAF15/RBM39 pathway (Chen et al., 2021). Another study used a matrix metalloproteinase 2 (MMP-2) promoter-luciferase reporter assay to demonstrate that the transcription of MMP-2 in K-RAS knockout fibroblasts was partially restored by transient expression of K-RAS4B but not K-RAS4A (Liao et al., 2003). This finding suggests that K-RAS4B has a greater metastatic potential than K-RAS4A, because tumour cells that express oncogenic RAS have a higher metastatic potential partially due to up-regulation of MMP-2 (Liao et al., 2003). Overall, both reports support a more tumorigenic role for K-RAS4B than K-RAS4A.

Interestingly, K-RAS4A shares similarities with H-RAS; both have been shown to induce lung tumours in wildtype and H-RAS knock-in mice (To et al., 2008). Since K-RAS presents mutations at the same regions in both splice variants, the vast majority affecting codon G12, some cancers may harbour mutations in one or even both isoforms simultaneously. Thus, blocking one isoform might not be enough to fully reduce the cell’s oncogenic potential. Oncogenic K-RAS4A has also been shown to induce lung carcinogenesis in mice (To et al., 2008), and a recent publication by the Barbacid group demonstrated that expression of K-RAS4A^G12V^ in mice that lack K-RAS4B is sufficient to promote metastatic lung adenocarcinomas (Salmon et al., 2021). These reports highlight K-RAS4A’s oncogenic potential, suggesting it could serve as a future therapeutic target.

Studies performed on patient samples have also supported different roles for each isoform. Abubaker and colleagues found an association between K-RAS4A overexpression and improved overall survival in patients with colorectal cancer, whereas overexpression of K-RAS4B was significantly associated with larger tumour size (Abubaker et al., 2009). The RAS oncogene is also involved in cell metabolism, and it was suggested that distinct RAS mutations might lead to variable metabolic dependencies (Kimmelman 2015). Recently, hexokinase 1 (HK1) was shown to be a K-RAS4A effector, which could impact on the tumours’ cells metabolism (Amendola et al., 2019).

In human K-RAS-mutant leukaemia cell lines and in acute myeloid leukaemia (AML) cells, K-RAS4A is also expressed, and Zhao and colleagues showed that cells harbouring mutations at the palmitoylation site of oncogenic K-RAS4A (i.e., palmitoylation-defective mutant K-RAS4A^G12D/C180S^) present a reduction in leukemogenicity potential. Unlike the results seen with mutations at the palmitoylation site of N-RAS (i.e., palmitoylation-defective mutant N-RAS^G12D/C181S^), palmitoylation-defective K-RAS4A could still induce leukaemia in mice (Zhao et al., 2015). The KIKK motif of K-RAS4A appears to impact on its transforming activity since mutations affecting both the palmitoylation site and the KIKK motif blocked oncogenic K-RAS4A from inducing leukaemia in mice (Zhao et al., 2015). These findings support a role for the different posttranslational modifications in RAS function and oncogenic potential.

The fact that both splice variants are identical in the region where most K-RAS oncogenic mutations occur suggests that previous reports of mutations in K-RAS may actually have uncovered mutations in both transcripts, not just in K-RAS4B. In addition, cancers harbouring K-RAS mutations may behave differently depending on which splice variant is predominantly affected, which could impact on therapy response. As K-RAS4A and K-RAS4B possess slightly different structures when in the GDP-bound state, with GDP-bound K-RAS4A presenting a more exposed nucleotide binding pocket than GDP-bound K-RAS4B (Chakrabarti et al., 2016), compounds developed to target this catalytic domain could also be considered as a means to differentiate between the oncogenic mutant variants. The recent FDA approval of Sotorasib or Lumakras (previously known as AMG 510, Amgen), a K-RAS^G12C^ inhibitor able to reduce K-RAS^G12C^ tumours (Canon et al., 2019; Hong et al., 2020), is a major breakthrough in RAS biology, since for many years RAS was considered an undruggable target. How efficient this drug is when comparing K-RAS4A^G12C^ versus K-RAS4B^G12C^ in different cancer types remains to be determined. It would be interesting to see the development of novel mutation- and splice variant-specific inhibitors in those cancers where both isoforms are simultaneously affected. Nevertheless, more analysis should be performed to better clarify if there are any significant differences between mutant K-RAS4A and mutant K-RAS4B in response to distinct therapies.

## Conclusion

K-RAS4B research has historically overshadowed that of K-RAS4A, suggesting that K-RAS4A is a minor variant. Nevertheless, the fact that K-RAS4A is evolutionarily conserved and binds distinct effectors at different affinities compared to K-RAS4B, in addition to the fact that K-RAS4A expression varies across tissue types, argue for a more important role than previously thought. Additional work is needed to unravel the different roles that each splice variant plays in normal versus tumours tissues. Such knowledge may help inform understanding of therapy resistance and improve disease management of cancer types with differential splice variant expression. Personalised medicine has exploited K-RAS-mutation-specific tumour differences for the development of mutation-selective anti-RAS strategies; thus, it could be beneficial to place K-RAS4A in the spotlight and perhaps achieve more selective cancer treatment strategies.
